# Development and Validation of a Stability-Indicating Capillary Electrophoresis Method for the Determination of Zolpidem Tartrate in Tablet Dosage Form with Positive Confirmation using 2D- and 3D-DAD Fingerprints

**DOI:** 10.3797/scipharm.1401-11

**Published:** 2014-02-27

**Authors:** Khaldun M. Al Azzam, Lee Kam Yit, Bahruddin Saad, Hassan Shaibah

**Affiliations:** 1 Faculty of Pharmacy, Isra University, 11622 Amman, Jordan.; 2 Pharmacy Program, Batterjee Medical College for Science and Technology (BMC), 21442 Jeddah, Kingdom of Saudi Arabia.; 3 School of Chemical Sciences, Universiti Sains Malaysia, 11800 Penang, Malaysia.

**Keywords:** Zolpidem tartrate, Capillary zone electrophoresis, Tablet dosage form, Stability indicating

## Abstract

The aim of the current study was to develop a simple, precise, and accurate capillary zone electrophoresis method for the determination of zolpidem tartrate in tablet dosage form. Separation was conducted in normal polarity mode at 25°C, 22 kV, using hydrodynamic injection for 10 s. Separation was achieved using a background electrolyte of 20 mM disodium hydrogen phosphate adjusted with phosphoric acid (85%), pH at 5.50, and detection at 254 nm. Using the above optimized conditions, complete determination took place in less than 3 min using amiloride HCl as the internal standard. The method was linear over the range of 3–1000 μg mL^−1^ with a correlation coefficient of 0.9999. Forced degradation studies were conducted by introducing a sample of zolpidem tartrate standard and pharmaceutical sample solutions to different forced degradation conditions, being neutral (water), basic (0.1 M NaOH), acidic (0.1 M HCl), oxidative (10% H_2_O_2_), temperature (60°C in oven for 3 days), and photolytic (exposure to UV light at 254 nm for 2 h). Degradation products resulting from the stress studies did not interfere with the detection of zolpidem tartrate and the assay can be considered stability-indicating.

## Introduction

**Fig. 1:**
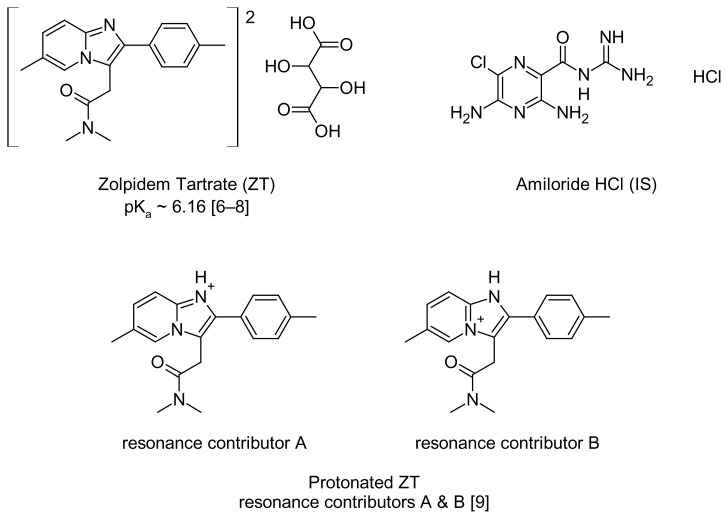
The chemical structures of the drug (ZT), amiloride HCl (IS), and the resonance form of the protonated ZT.

Zolpidem tartrate (ZT), *N*,*N*-dimethyl-2-[6-methyl-2-(4-methylphenyl)imidazo[1,2-*a*]pyridin-3-yl]acetamide 2,3-dihydroxybutanedioate (2:1) [1] (Fig. 1), an imidazopyridine hypnotic that was introduced into clinical practice in the United States in 1992, is the most commonly prescribed drug against insomnia [2]. It has been reported as a new hypnosedative agent to treat short-term insomnia [1, 3, 4]. Insomnia is a distressing condition that affects not only the ability to sleep at night, but also the ability to function effectively during one’s desired waking time. Symptoms associated with insomnia include difficulty in initiating sleep, or even waking up early without being able to sleep again. Risk factors for insomnia include increasing age, medical comorbidities, psychiatric illness, impaired social relationships, circadian rhythm disturbances, and poor sleep hygiene [5]. ZT is clinically effective, safe, and well-tolerated. Moreover, it has a favorable pharmaco-kinetic profile for use as a hypnotic where its absorption and elimination is rapid [2].

Recently, there has been an increased tendency towards the development of stability-indicating assay methods, using the stress testing approach as specified under the International Conference on Harmonization ((ICH) guidelines Q1A (R2, 1995)) [10]. It requires that the fully validated analytical test procedures should be stability-indicating and carried out under harsh conditions to give information on a drug’s inherent stability [11]. Its importance implies providing evidence on the quality of the bulk drug when subjected to external environmental conditions such as temperature, pH, and light. The data obtained facilitate storage conditions such as shelf life to be established as well as re-testing periods [12].

Several analytical methods for the determination of ZT have been reported in pharmaceutical formulations and plasma using high-performance liquid chromatography (HPLC) whether coupled with UV detection in *in-process* control synthesis samples [4], in rat serum [13], to be stability-indicating in pharmaceutical formulations [14], with fluorescence in human plasma samples [15, 16], mass spectrometry in oral fluid liquid [3], in pharmaceutical formulation [17], or in hair [18]. Gas chromatography coupled with mass spectrometry (GC-MS) in postmortem specimens [19] and capillary electrophoresis (CE) coupled with fluorescence detection in urine [20] have also been reported.

These methods either use expensive mass spectrometers [17, 21, 22] or have a long separation time (4–8 min) [4, 13–16, 20] that is not suitable for the analysis of a large number of samples.

CE is a powerful analytical technique that is carried out in narrow-bore capillaries under the influence of an external electric field [23], thanks to its numerous benefits such as its super resolution, speed of analysis, versatility, high efficiency, its unique separation mechanism due to its flexibility to switch from one mode of separation to another, and the ease to be coupled with many kinds of detectors [24]. Additionally, CE has shown great potential in the analysis of inorganic ions, biopolymers, as well as drugs.

The aim of the current work was to develop and validate a rapid, simple, inexpensive, and selective stability-indicating assay CE method, systematically optimized and validated, and for the first time, to be applied in the routine quality control analysis of ZT in tablets as requested by the International Conference on Harmonization (ICH) guidelines ((ICH) guidelines Q1A (R2, 1995) & Q1B (1996))) [24, 25]. The developed method will be applied to the determination of ZT not only in the active pharmaceutical ingredient (API), but also in pharmaceutical tablets under different stressed conditions, as well as to the analysis of pharmaceutical tablets.

## Experimental

### Chemicals and Reagents

The ZT and amiloride hydrochloride (IS) standards were kindly donated by HIKMA Pharmaceutical Company, Amman, Jordan. Phosphoric acid (85%), sodium hydroxide, disodium hydrogen phosphate, citric acid, boric acid, and tris(hydroxymethyl) amino-methane were purchased from Sigma–Aldrich (St Louis, USA). Hydrochloric acid (37%) and hydrogen peroxide (31%) were purchased from Merck (Darmstadt, Germany). The commercial pharmaceutical preparation in the form of tablets was purchased from local pharmacies. Deionized water was produced using a Milli-Q system (Millipore, Bedford, USA), and was used throughout for the preparation of solutions.

### Instrumentation and Electrophoretic Conditions

Separations were conducted on a HP^3D^CE capillary zone electrophoresis system (Agilent Technologies, model G1600A, Waldbronn, Germany). The unit was equipped with a photodiode array (PDA) detector. Data acquisition was performed with ChemStation Software. An uncoated bare fused-silica capillary 50 μm i.d× 40 cm (detection length, 8.5 cm from the outlet end of the capillary) from Agilent Technologies was used. The new capillary was conditioned by flushing for 20 min with 1 M NaOH, 10 min with 0.1 M NaOH, and 10 min with water. Between injections, it was preconditioned with 0.1 M NaOH, and then purified water, followed by the background electrolyte (BGE), each for 3 min between the runs. Samples and standards were injected hydrodynamically at 50 mbar for 10 s under the following conditions: voltage, 22 kV (normal polarity); capillary temperature, 25°C; detector wavelength, 254 nm; and BGE, 20 mM disodium hydrogen phosphate – adjusted with phosphoric acid (85%); pH, 5.50. At the end of the day, a final 5 min washing with water, and 1 min from the empty vial were performed. All standards, sample solutions, BGE, and NaOH solution were filtered through a 0.2 μm regenerated cellulose membrane filter (Germany) using an Agilent solvent filtration kit. The ultrasonic water bath sonicator, model 28X (ULTRAsonik, Ney Dental, Yucaipa, California) was used for sonication. An Orion pH meter model EA 940 (Orion Research, Cambridge, USA) was used for pH measurements. Analytical balance model AY 220 (Shimadzu Corp., Japan), UV irradiator model USHIO Optical Modulex, America Inc., (Cerritos, Ave, Cyres, California), and an electric oven (Memmert W 8540 Schwabach, Germany) were also used throughout the study.

### Preparation of Standard Solutions

Stock solutions of ZT (2000 μg mL^−1^) and amiloride hydrochloride as internal standard (1000 μg mL^−1^) were prepared in water. The stock solutions were used to prepare calibration standards. Working solutions for the calibration curve were prepared by serially diluting the stock solution with water after spiking with the internal standard to a final concentration of 100 μg mL^−1^. All solutions were kept in the refrigerator and under light protection when not in use.

### Pharmaceutical Sample Preparation

Sobrium tablets containing 10 mg of ZT were accurately weighed, crushed together, and finely powdered. An accurately weighed amount of the powder equivalent to 2.5 mg of ZT was transferred and dissolved with the aid of water, sonicated using an ultrasonic water bath for 5 min, and then diluted to 25 mL with water after spiking with the internal standard to a final concentration of 100 μg mL^−1^. A small portion of the sample was filtered through a regenerated cellulose membrane (0.2 μm). This solution was introduced to the CE system for the separation. All solutions were kept in the refrigerator and under light protection when not in use.

### Forced Degradation Studies

Forced degradation studies were conducted in accordance with the ICH guidelines ((ICH) guidelines Q1A (R2, 1995) & Q1B (1996)) [25] by introducing sample and active pharmaceutical ingredient (API) tablets of ZT solutions to different forced degradation conditions; namely, neutral (water), basic (0.1 M NaOH), acidic (0.1 M HCl), oxidative (10% H_2_O_2_), photolytic (exposure to UV light at 254 nm for 2 h), and thermal (exposure to heat at 60°C for 3 days consecutively). The neutral, acidic, basic, and oxidative solutions were refluxed at 80°C for 6 h and sampled at 2 h intervals. The peak purity of ZT in the generated electropherograms was checked using a photodiode array detector.

The specificity of the method was determined by checking the interferences of the tablet placebo with the ZT peak and was assessed by preparing the placebo according to the manufacturer’s practice. The Sobrium ZT tablet and placebo tablet consisted of starch, lactose monohydrate, sodium starch glycolate, sodium lauryl sulfate, colloidal silicon dioxide, magnesium stearate, lactose monohydrate, hydroxypropylmethyl cellulose, polyethylene glycol, titanium dioxide, and iron oxide yellow [26]. The placebo concentration in the synthetic samples was fixed at 6.0 mg mL^−1^ and then subjected to the CE analysis under the same conditions.

## Results and Discussion

### Optimization of Separation Conditions

The CE method was optimized to develop a stability-indicating assay that was able to resolve the degradation products from the ZT peak. Electrophoretic conditions were selected after a full investigation of the different types of BGE compositions such as citrate, borate, and tris(hydroxymethyl)aminomethane, but all of these attempts failed to produce an acceptable peak shape.

Preliminary trials were done using different types of BGE and even different modes of separations; namely, micellar electrokinetic chromatography (MEKC), (MEKC conditions: BGE, 20 mM disodium hydrogen phosphate – adjusted with phosphoric acid (85%); pH 3.50, containing 150 mM sodium dodecyl sulfate (SDS); capillary temperature, 25°C; voltage, 20 kV (negative polarity); and injection time, 10 s). Using the previous MEKC conditions, we were able to resolve the degradation products from the ZT peak. Extra attention has been paid to simplify the BGE compositions and to test the ability of this BGE to separate the degradation products without surfactant’s aid and the results were promising, thus we have focused on the current BGE for further optimization of the separation of the degradation products from the ZT peak after exposing a small portion of the API to different stressed conditions. The migration time was obtained (< 3 min) that is important for quality control routine analysis.

ZT is a basic drug with a pK_a_ value of about 6.16 [6–8]. At low pH (< 5.6), it becomes positively charged and moves towards the cathode in the same direction with the electroosmotic flow (EOF) [20]. The amidine structure in ZT possesses enhanced basicity due to resonance stabilization of the protonated cation once it is in acidic medium. Studies demonstrate that alkyl substituents enhance pK_a_ (~12), while the phenyl group decreases the value to about 8. Additionally, the unsaturated vinyl group should also act to decrease the value. Protonation yields a resonance-stabilized cation incorporating the contributors A and B (Fig. 1). From Figure 1, resonance contributor B is the major one as a result of resonance stabilization by the aromatic pyridinium ion, which also should be a driving force for protonation [9]. In the current study, the effect of the buffer pH over the range 4.0–9.5 on the migration time using disodium hydrogen phosphate, adjusted with concentrated phosphoric acid (85%), was tested. Therefore, a pH of 5.50 was selected since a peak with good shape and reasonable migration time was obtained (< 3 min). It was noticed that at a high pH, the migration time of the analyte slightly improved. This is attributed to that fact that as pH increases, EOF will increase accordingly and thus have a faster analysis time.

The effect of the concentration of disodium hydrogen phosphate (20–50 mM) at a fixed concentration of phosphoric acid (85%) was investigated at the constant pH 5.50. A high buffer concentration reduces the EOF due to the increase in the number of ions and thus reduces solute mobility, due to increased drag caused by the counter-migration of the densely packed counterions, and thus results in a reduction in the peak efficiency and a longer migration time and vice versa. Besides, higher buffer concentrations are more conductive, draw higher current, and therefore produce more heat than the dilute buffer solution [27]. Therefore, 20 mM of disodium hydrogen phosphate was used for the next measurements.

The effect of capillary temperature over the range (15–30°C) on the peak shape and migration time was studied. As temperature increased, the migration time of the analyte decreased because the viscosity of the BGE decreased, thus having stronger EOF and faster analysis time accordingly. The viscosity of fluid is always inversely proportional to temperature. Moreover, it is important to take into consideration that high temperatures in the capillary will lead to the generation of Joule heating, which will eventually cause peak broadening. As a compromise between the speed of analysis and peak shape, 25°C was adopted, although higher temperatures will give faster migration times.

The effect of applied voltage was investigated by varying the voltage from 18–26 kV. As voltage increases, migration time will decrease due to the increase in EOF velocity and thus shorter migration time. However, the generation of Joule heating causes parabolic flow which in turn may affect the resolution and peak efficiency upon increasing the voltage [27, 28]. On the other hand, at low voltages, molecular diffusion is the main cause of band broadening [27]. Therefore, 22 kV was chosen for the rest of the studies as it gives a good peak shape and reasonable migration time.

**Fig. 2:**
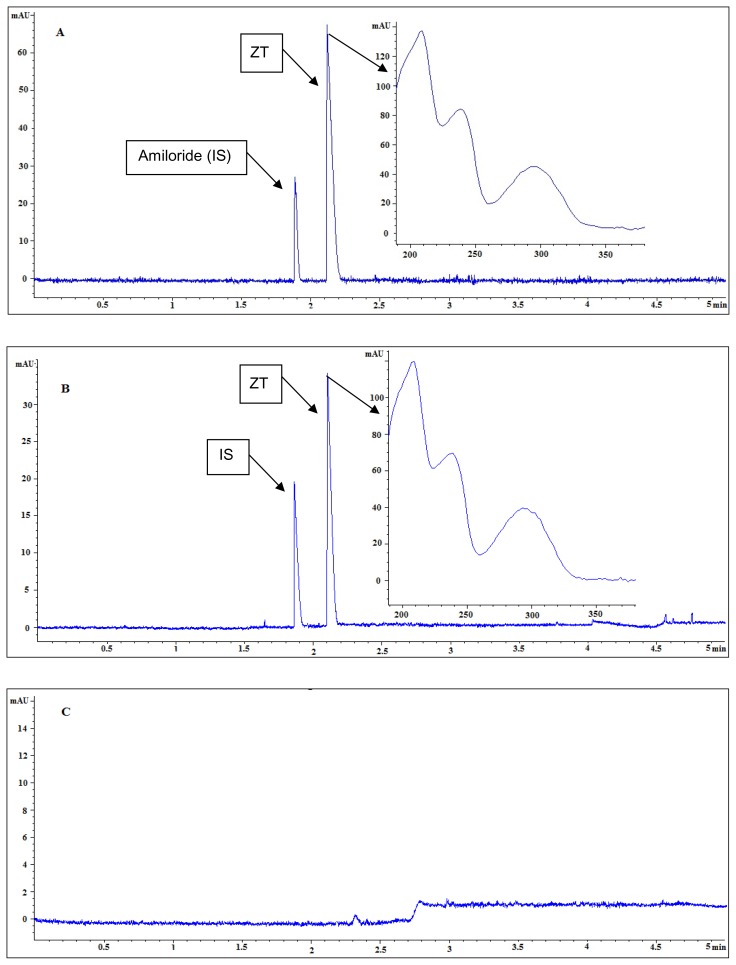
Electropherograms obtained from the injection of ZT standard (250 μg mL^−1^) (A); Sobrium tablet (100 μg mL^−1^) (B); and tablet placebo (C). Please refer to text for CE conditions.

The effect of injection time was studied over the range (5–20 s) at 50 mbar. As expected, as the injection time was increased, peak area increased. Moreover, peak broadening was observed. Therefore, an injection time of 10 s was used for the rest of the study. The adopted electrophoretic conditions are summarized in Table 1, while Figure 2(A) shows the typical electropherogram. It is clear that the selected electrophoretic conditions provide good peak shape.

### Validation of Analytical Method

Validation was conducted based on the ICH guidelines ((ICH) guidelines Q1A (R2, 1995)) [10]. The importance of using IS has been reported previously to reduce the injection-related impression and to achieve better reproducibility and a greater control over the sample amount introduced. Thus, the use of an IS in quantitative analysis is generally preferred [29]. Therefore, amiloride HCl as IS was chosen in the current work as it is a basic compound and is positively charged (p*K*_a_ 8.7) at the studied pH value [30].

Moreover, the suitability of amiloride as IS is evident because it resolved well from the analyte peak and the degradation products.

#### Calibration Curve, Limits of Detection, and Quantitation

Nine standard solutions of ZT were prepared in the concentration range (3–1000 μg mL^−1^) and were introduced in order to establish the linearity. The calibration curve was constructed by plotting the relative corrected peak area (y) versus the ZT concentration (x) in μg mL^−1^. The ZT calibration curve showed excellent linearity with the following regression equation:

$$\text{y}=0.015\text{x}+0.103,\;{\text{r}}^{2}=0.9999$$

The LOD of ZT, which was calculated as the sample concentration was introduced to yield a signal-to-noise ratio (S/N) of 3, was found to be 0.81 μg mL^−1^. While the LOQ was calculated, the sample concentration was introduced to yield a signal-to-noise ratio (S/N) of 10. The LOQ was found to be 2.70 μg mL^−1^ (Table 2).

The sensitivity of the newly developed CE method is inferior when compared to the reported HPLC-UV methods [4, 14], HPLC-fluorescence [15], or to LC-MS/MS [3, 17, 31] (Table 2). However, the analysis time of the new CE method is slightly faster (< 3 min compared to ~5–8 min) in the HPLC reports [4, 13–17] or compared to the GC report (~5.28 min) [18]. Although the sensitivity is inferior compared to the reported methods, it possesses adequate sensitivity for the analysis of the active ingredient in the formulations.

#### Precision

The precision of the developed method was examined by performing intra- and inter-day precision and was expressed as relative standard deviations (RSD). Intra-day precision (repeatability) was performed by introducing the standard solutions of three concentrations (10, 500, and 1000 μg mL^−1^). Each was introduced to the CE system three times (n = 9). In all cases, the % RSD of migration time, peak area, corrected peak area, ratio of corrected peak area, and ratio of peak area were less than 2.43, 3.45, 4.91, 3.11, and 4.69%, respectively (Table 2).

Inter-day precision (intermediate precision) was assessed by introducing the same three concentrations mentioned earlier for inter-day precision over 3 consecutive days (n = 27). Moreover, the RSD for migration times, peak area, corrected peak area, ratio of corrected peak area, and ratio of peak area were less than 3.04, 4.65, 4.94, 5.98, and 5.76%, respectively (Table 2). Results obtained from the determination of repeatability and intermediate precision, expressed as RSD (%), indicate the good repeatability of the newly developed method for the determination of ZT in tablet dosage form.

#### Accuracy

The accuracy test was conducted by adding known amounts of ZT to the placebo preparation. It was carried out at three concentrations (25, 500, and 900 μg mL^−1^) and each was introduced to the CE thrice (n = 9). Accurately weighed amounts of the placebo were dispersed in the standard solutions at a fixed concentration of 6.0 mg mL^−1^. Afterward, the actual and measured concentrations were compared. The values obtained (100.09, 99.91, and 103.63%) proved the good accuracy of the newly developed method for the determination of ZT in tablet dosage form (Table 2).

#### Specificity

The specificity of the method was assessed by checking the interferences of the placebo with the analyte. No peaks were obtained for the ZT placebo or no such other peaks were even eluted in the same ZT migration time (Fig. 2(C)). Moreover, the selectivity of the developed method was examined by subjecting the ZT standard and the tablet formulation to different degradation mediums as mentioned in section 2.5. There were no such interferences of any peak of the degradation products with the ZT peak. The degradation solutions were introduced to the CE analysis under the adopted conditions. Additionally, the peak purity of ZT was checked using the PDA detector equipped with the CE unit. Absorption spectra were compared at the upslope, apex, and downslope of the ZT peak. The three overlaid UV spectra were the same, indicating the selectivity and peak purity of the developed method. Thus the peak purity was found to be satisfactory under the forced degradation studies conducted.

**Fig. 3:**
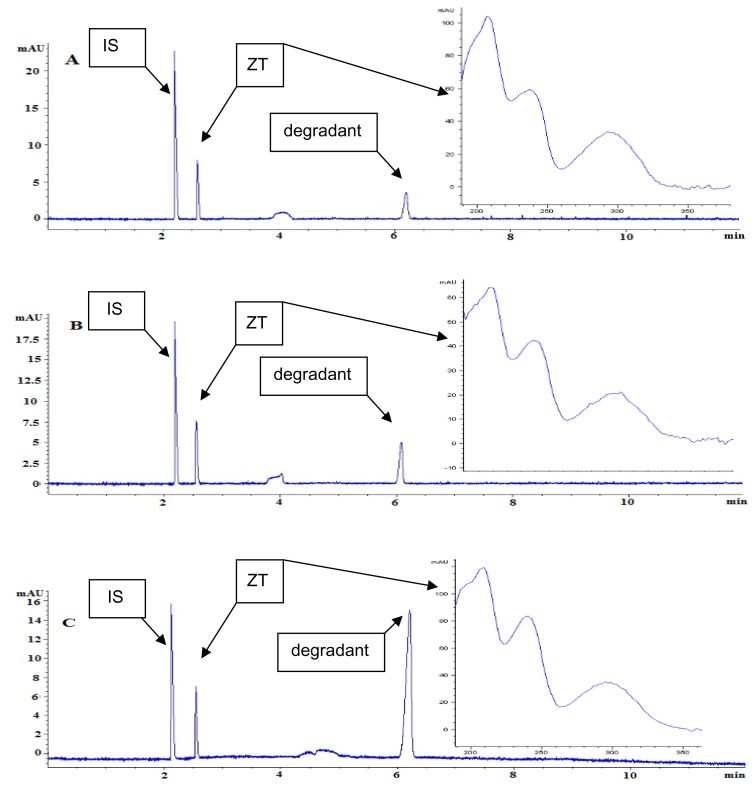
Typical electropherograms obtained. Samples were heated in 0.1 M NaOH for (A) 2 h, (B) 4 h, and (C) 6 h. Please refer to text for CE conditions.

Comparing the electropherograms (Fig. 3), in the forced degradation studies, it obviously elucidates the disappearance of ZT and the formation of degradation products under basic forced conditions, suggesting that the assay is stability-indicating as well as capable of separating ZT in the presence of degradation products and confirming the absence of other co-eluting substances.

**Fig. 4:**
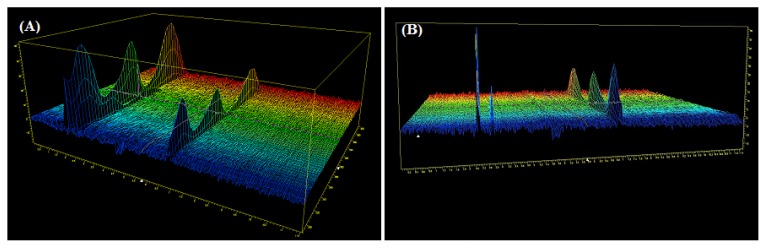
Method specificity (view A & B). CE-DAD analysis of the ZT Sobrium tablet after refluxed at 80°C in 0.1 M NaOH for 6 hours under the optimized electrophoretic conditions (please refer to Table 1 for the CE conditions). The concentration of ZT is 100 μg mL^−1^

Results for the peak purity obtained from the diode array detector under the optimized electrophoretic conditions confirmed that the two peaks (ZT peak & degradant) were homogenous and pure in all stress samples analyzed that showed this degradant, which revealed that no such additional peaks were co-eluting with the ZT peak. Moreover, FDA guidance indicates that well-separated peaks, with resolution Rs >2 between the peak of interest and the closest eluted peak, are important for reliable quantification. Both of the two peaks (ZT & degradant) met this requirement, and even visibly confirmed as shown in Fig. 4.

#### Analysis of Pharmaceutical Tablets

The newly developed method was applied for the determination of ZT in a commercially available ZT tablet (Sobrium 10 mg). Assay results yielded 103.09 ± 0.80% of the label claim for Sobrium, (there were two preparations and each was introduced thrice to the CE system). Good agreement between the total value as claimed by the manufacturer and the developed CE method was obtained. Figure 2(B) shows the typical electropherogram of the tablet dosage form.

#### Stressed Degradation

Generally, the results showed that ZT in tablet dosage form was more stable compared with the standard solution under elevated temperature in water, 0.1 M HCl, 0.1 M NaOH, and under temperature (60°C in oven for 3 days), while it was not stable under UV light exposure. This behavior may be attributed to the effect of the tablet excipients which may mask ZT’s active ingredient from complete exposure to the forced degradation conditions. Under the alkaline conditions, ZT in standard solution was almost degraded compared with the tablet dosage form, and the amounts of ZT recovered were 16.47 and 91.58%, respectively (Table 3).

Interestingly, it was noticed that the color of both dry powder and solution of the tablet dosage form changed to slightly yellowish upon exposure to the UV light compared with the ZT standard solution. This may interpret the low recovery values obtained between the tablet formulation and the standard form. In the current study, extra efforts have been made to correlate the results of the forced degradation. The mechanism of photo-degradation involves the breaking and/ or oxidation of the tertiary amide moieties or imidazopyridine in the zolpidem molecule. Three degradation products during that process can arise: oxozolpidem, zolpaldehyde, and zolpyridine. These degradation products could affect the chemical, toxicological, and pharmaceutical properties of the pharmaceutical dosage form. Thus, the determination of degradation products is deemed necessary, due to possible changes in properties that may occur [1].

**Fig. 5:**
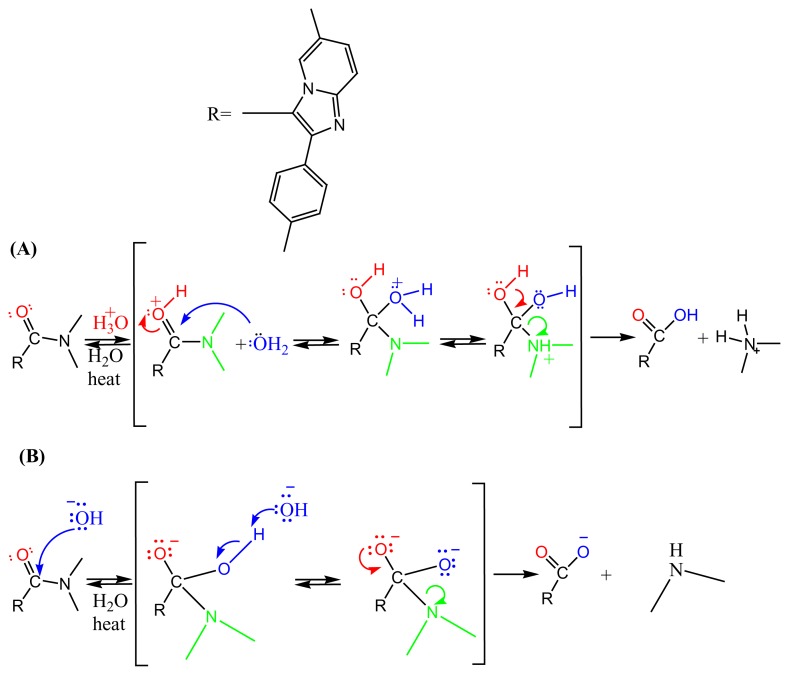
Proposed mechanisms for the reactions of ZT upon (A) acid hydrolysis and (B) base hydrolysis.

ZT undergoes slow degradation in acidic solutions, due to the instability of the tertiary amide moiety present in the ZT structure. Products of acid hydrolysis are zolpacid and dimethylamine based on the proposed mechanism (Fig. 5(A)). Other than being a degradation product, zolpacid is also recognized as an impurity related to zolpidem synthesis. Major degradation was observed in the alkaline medium and the ZT peak was degraded up to 10.69%. The major impurity peak was observed at about 6 min (Fig. 3(C)). Major products of that basic hydrolysis are carboxylate and amine (Fig. 5(B)).

It can be declared again that no interferences either from excipients present in the tablet formulation or from the other peaks generated by the stress degradation interfered with the ZT peak (Fig. 3), indicating the specificity of the newly developed method and its suitability to be used for quality control routine work.

## Conclusion

A simple, accurate, specific, and precise stability-indicating CE method for the determination of ZT in API and dosage forms was developed and validated. The new method has been validated in accordance with ICH guidelines ((ICH) guidelines Q1A (R2, 1995) & Q1B (1996)) [10, 25]. Good analytical performance with regards to linearity, accuracy, and precision was achieved. It was found that the drug (solution & powder) was stable for both the standard and tablet dosage form when exposed to thermal (exposure to heat at 60°C for 3 days consecutively), neutral, acidic, and oxidative solutions when refluxed at 80°C for 2 hours. However, the drug was rather unstable under the degradation stress of basic medium when refluxed at 80°C for 2 hours and under UV irradiation at 254 nm. The short run time for ZT and internal standard in less than 3 minutes has proven that the analysis is rapid, thus it is recommended to be adopted as a quality control protocol in pharmaceutical industries for the analysis of large numbers of samples. The simplicity of the newly developed method allows for its application in laboratories that do not have the expensive analytical instruments such as LC-MS or GC-MS. Moreover, the later methods are considered complicated, time consuming, and costly compared with the CE method. Higher separation efficiency and the minimization of the use of solvents are other inherent features of the CE methods. Additionally, the CE method did not suffer interference by the tablet formulation excipients, since no other peaks occurred in the same ZT retention time.

## Figures and Tables

**Tab. 1:** Adopted CE operating conditions

Background electrolyte	20 mM disodium hydrogen phosphate-concentrated phosphoric acid; pH 5.50.
Applied voltage	22 kV (normal polarity)
Sample injection	10 s hydrodynamic injection
Capillary temperature	25°C
Fused silica capillary	50 μm i.d × 40 cm (detection length), 8.5 cm from the outlet end of the capillary
Detection wavelength	254 nm

**Tab. 2:** Results of intra-day (repeatability), inter-day precision (intermediate precision), accuracy, and the comparison of the developed method with other reported methods for the determination of ZT.

Am*	RSD (%)									

	MT	PA	CPA	RCPA	RPA	Am*	% Recovery ± SD (n = 9)	LOD*	LOQ*	Method [Reference]
Intra-day precision (n = 9)#										

10	2.43	3.45	4.91	3.11	3.50	25	100.09±0.77	0.81	2.70	Current work
500	1.60	3.12	3.96	2.68	4.69	500	99.91±0.86	0.1	–	HPLC-UV [4]
1000	1.05	1.87	1.28	2.15	1.39	900	103.63±0.99	0.026	–	HPLC-UV [14]
								0.0000015	–	HPLC-FL [15]
								0.0002	–	LC-MS/MS [3]
								0.0001	–	LC-MS/MS [17]
								0.00017	–	LC-MS/MS [30]

Inter-day precision (n = 27)#										

10	2.90	2.68	4.12	5.56	4.76					
500	3.04	4.65	4.94	5.98	5.76					
1000	2.28	1.70	1.93	3.80	3.63					

* [μg mL^−1^];

# No. of introductions to the CE system (three preparations for each concentration);

Am…Amount; MT…Migration time; PA…Peak area; CPA…Corrected peak area; RCPA…Ratio of corrected peak area; RPA…Ratio of peak area; HPLC-UV…High-performance liquid chromatography coupled with UV detector; HPLC-FL…High-performance liquid chromatography coupled with fluorescence detector; LC-MS/MS…Liquid chromatography–mass spectrometry.

**Tab. 3:** Results for the determination of the ZT standard and tablet dosage form when subjected to different stressed conditions

Stress Condition	% Recovery ± SD	

	Standard	Pharmaceutical Formulation
Water		
2 h	97.35 ± 0.39	103.36 ± 1.02
4 h	85.58 ± 0.39	97.58 ± 0.77
6 h	76.91 ± 0.38	96.69 ± 0.38
10% H_2_O_2_		
2 h	95.80 ± 0.67	94.24 ± 0.77
4 h	83.58 ± 0.77	81.13 ± 0.67
6 h	76.69 ± 0.77	77.36 ± 0.77
HCl 0.1 M		
2 h	94.25 ± 0.39	95.14 ± 1.15
4 h	82.69 ± 0.77	88.24 ± 1.02
6 h	72.02 ± 0.77	75.13 ± 1.15
NaOH 0.1 M		
2 h	16.47 ± 1.02	91.58 ± 0.39
4 h	14.69 ± 0.38	85.36 ± 0.77
6 h	10.69 ± 0.77	77.58 ± 1.02
UV exposure (254 nm) 2 h		
Solution	81.13 ± 0.67	62.78 ± 1.10
Dry powder	92.47 ± 1.02	78.18 ± 0.37
Oven (3 days)		
Solution	91.76 ± 0.95	95.06 ± 0.61
Dry powder	93.80 ± 0.67	95.82 ± 0.38
